# Investigation of Novel Small Molecular TRPM4 Inhibitors in Colorectal Cancer Cells

**DOI:** 10.3390/cancers13215400

**Published:** 2021-10-28

**Authors:** Paulina Stokłosa, Anna Borgström, Barbara Hauert, Roland Baur, Christine Peinelt

**Affiliations:** Institute of Biochemistry and Molecular Medicine, University of Bern, 3012 Bern, Switzerland; paulina.stoklosa@ibmm.unibe.ch (P.S.); anna.borgstroem@ibmm.unibe.ch (A.B.); barbara.hauert@ibmm.unibe.ch (B.H.); roland.baur@ibmm.unibe.ch (R.B.)

**Keywords:** TRPM4, ion channels, small molecule inhibitors, patch clamp

## Abstract

**Simple Summary:**

Transient receptor potential melastatin 4 (TRPM4) ion channel malfunction or aberrant expression is implicated in many diseases, including different cancers and cardiovascular diseases. Currently, there is a need for specific and potent TRPM4 inhibitors. They would allow to study the role of TRPM4 in disease models and to validate it as a potential target in therapies, including anti-cancer therapy. In colorectal cancer (CRC), TRPM4 is upregulated, and its conductivity plays a role in the regulation of viability and cell cycle of CRC cells. In this study, we tested three novel TRPM4 inhibitors, CBA, NBA, and LBA, in CRC cells. In HCT116 cells, we show that NBA inhibits TRPM4 currents in the micromolar range and alters proliferation and cell cycle. Furthermore, NBA decreases the viability of Colo205 cells. This makes NBA a promising candidate for further evaluation as a specific TRPM4 inhibitor in other cellular systems and disease models.

**Abstract:**

(1) Background: Transient receptor potential melastatin (TRPM4) ion channel aberrant expression or malfunction contributes to different types of cancer, including colorectal cancer (CRC). However, TRPM4 still needs to be validated as a potential target in anti-cancer therapy. Currently, the lack of potent and selective TRPM4 inhibitors limits further studies on TRPM4 in cancer disease models. In this study, we validated novel TRPM4 inhibitors, CBA, NBA, and LBA, in CRC cells. (2) Methods: The potency to inhibit TRPM4 conductivity in CRC cells was assessed with the whole-cell patch clamp technique. Furthermore, the impact of TRPM4 inhibitors on cellular functions, such as viability, proliferation, and cell cycle, were assessed in cellular assays. (3) Results: We show that in CRC cells, novel TRPM4 inhibitors irreversibly block TRPM4 currents in a low micromolar range. NBA decreases proliferation and alters the cell cycle in HCT116 cells. Furthermore, NBA reduces the viability of the Colo205 cell line, which highly expresses TRPM4. (4) Conclusions: NBA is a promising new TRPM4 inhibitor candidate, which could be used to study the role of TRPM4 in cancer disease models and other diseases.

## 1. Introduction

Imbalances in intracellular calcium (Ca^2+^) signaling contribute to cancer hallmarks, such as increased proliferation, higher motility, and reduced ability to undergo apoptosis [1]. Transient receptor potential melastatin 4 (TRPM4) belongs to the superfamily of widely expressed TRP channels. TRPM4 is a calcium-activated non-selective (CAN) channel that conducts monovalent cations, mainly sodium (Na^+^), without significant permeation to Ca^2+^. However, Na^+^ conductivity via TRPM4 contributes to the depolarization of the cell membrane, thereby reducing the driving force for Ca^2+^ entry through store-operated Ca^2+^ entry (SOCE), which is the main Ca^2+^ entry pathway in non-excitable cells, including cancer cells [2,3,4,5,6].

TRPM4 aberrant expression or malfunction have been linked to cardiovascular diseases, immune cell functions, and different types of cancer [4,6,7,8,9,10,11,12,13,14,15,16,17,18]. More than 20 genetic variants of TRPM4 have been described in patients with cardiac conduction dysfunctions, such as progressive conduction block or congenital atrioventricular block (AVB) [16,17,19]. Some of these pathological TRPM4 variants lead to loss or gain of expression and function [10,17,18]. Furthermore, TRPM4 is involved in the neurogenerative process of multiple sclerosis [20]. Recently, TRPM4 has emerged as a new potential player in carcinogenesis. TRPM4 protein expression is upregulated in prostate cancer (PCa) in comparison to benign glands. Furthermore, high TRPM4 expression has been associated with an unfavorable prognosis for PCa patients [6,14]. In PCa cells, TRPM4 alters Ca^2+^ signaling and contributes to the regulation of proliferation, migration, and invasion through upregulation of β-catenin oncogene signaling and its nuclear localization [6,21,22,23]. In breast cancer, TRPM4 was shown to be upregulated in comparison to normal breast tissue, and its expression was correlated with higher cancer stage and lymph node status. Furthermore, TRPM4 mRNA expression was associated with estrogen response and epithelial–mesenchymal transition (EMT) gene sets [24]. In addition, in diffuse large B cell lymphoma (DLBCL), TRPM4 expression was increased in comparison to normal B cells and was associated with a poor prognosis for the patients [25]. Moreover, in cervical cancer cells, TRPM4 regulates proliferation [26].

We recently described the role of TRPM4 in colorectal cancer (CRC). Immunohistochemical analysis of tissue microarrays from 379 CRC patients revealed that TRPM4 is highly expressed in tumor buds, which are clusters of up to five cells that detach from the original tumor site. Moreover, high TRPM4 expression was correlated with high numbers of tumor buds, as well as an infiltrative pattern of the tumor border. Both of these features correlate with an increased frequency of blood and lymphatic vessel invasion, as well as lymph node metastasis, which ultimately leads to an increased probability of disease reoccurrence and cancer-related death [11,27,28]. In addition, it was shown that TRPM4 is responsible for Ca^2+^-activated Na^+^ conductivity in CRC cell line, HCT116. Moreover, Na^+^ conductivity contributed to the regulation of CRC cells’ functions. Our results showed that the abolishment of TRPM4-mediated current resulted in reduced cell viability and induced a shift in the cell cycle. This effect was observed not only when TRPM4 protein was absent but also when a non-conducting channel was expressed. This suggests that TRPM4 conductivity is responsible for observed changes in cell viability and cell cycle [11].

**Figure 1:**
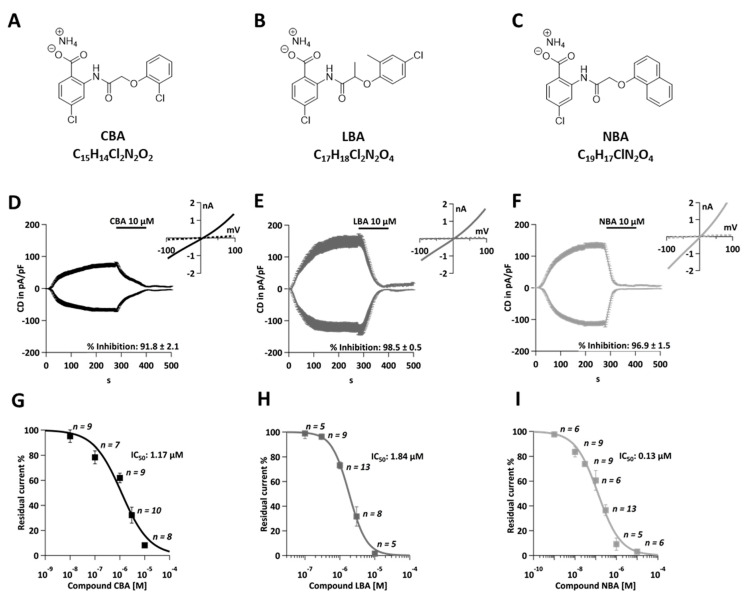
Novel TRPM4 inhibitors block TRPM4 currents in HCT116 cells. (**A**) Chemical structure of CBA. (**B**) Chemical structure of LBA. (**C**) Chemical structure of NBA. (**D**) Whole-cell patch clamp data from HCT116. Currents were evoked with 10 µM Ca2^+^ in the patch pipette and normalized to cell size. Current density (CD), displayed as mean +/− SEM, is plotted versus time and 10 µM CBA was applied from 280–400 s. Inset: IV curves at *t* = 276 s and *t* = 396 s (*n* = 8). (**E**) Same as (**D**) for LBA (*n* = 5). (**F**) Same as (**D**) for NBA (*n* = 6). (**G**) Average TRPM4 CD +/− SEM was plotted against CBA concentrations (*n* = 9 for 0.01 µM, *n* = 7 for 0.1 µM, *n* = 9 for 1 µM, *n* = 10 for 3 µM, and *n* = 8 for 10 µM). Dose–response curve was fitted with a Hill equation and IC_50_ value was 1.84 µM. (**H**) Average TRPM4 CD +/− SEM was plotted against LBA concentrations (*n* = 5 for 0.1 µM, *n* = 9 for 0.3 µM, *n* = 13 for 1 µM, *n* = 8 for 3 µM, and *n* = 5 for 10 µM). Dose–response curve was fitted with a Hill equation and IC_50_ value was 1.84 µM. (**I**) Average TRPM4 CD +/− SEM was plotted against NBA concentrations (*n* = 6 for 0.001 µM, *n* = 9 0.01 µM, *n* = 9 for 0.03 µM, *n* = 6 for 0.1 µM, *n* = 13 for 0.3 µM, *n* = 5 for 1 µM, and *n* = 6 for 10 µM). Dose–response curve was fitted with a Hill equation and IC_50_ value was 1.84 µM.

The above findings suggest that TRPM4 could be a potential drug candidate for anti-cancer therapy, but it has not been validated as such, partially due to the lack of potent and selective inhibitors. Until now, 9-phenantrol has been the most commonly used TRPM4 inhibitor [29,30]. However, 9-phenantrol exhibits low potency and selectivity towards TRPM4, which limits its application [31]. Other known TRPM4 inhibitors include flufenamic acid (FFA) and glibenclamide, but they act weakly on TRPM4 and show a number of off-target effects [13,32,33]. This shortage of specific and selective inhibitors supports the need for the development and validation of novel TRPM4 blockers (please see Figure 1A–C for chemical structures). We recently tested a new TRPM4 inhibitor, so-called compound 5 or CBA, in human PCa cell line LNCaP with the whole-cell patch clamp technique [19]. CBA inhibited TRPM4 currents in LNCaP cells, with an IC_50_ of 1.1 ± 0.3 µM, which was consistent with the data from the overexpression system. Furthermore, CBA showed good selectivity over other TRP channel family members (TRPV1, TRPV3, TRPV6, TRPM5, TRPM7, and TRPM8) [19]. Moreover, two other CBA derivatives were tested in PCa cells. LBA and NBA also showed strong inhibitory effect on TRPM4 currents with IC_50_ values of 0.74 ± 2.0 µM and 0.16 ± 2.4 µM, respectively, in LNCaP cells [34]. All three inhibitors, CBA, LBA, and NBA, show low cytotoxicity, making them useful for longer incubations [19]. Additionally, these inhibitors were tested in cellular assays of androgen insensitive PCa cells (DU145), but the TRPM4 blockers failed to specifically alter any of the analyzed cell functions, such as proliferation, viability, or migration. One possible explanation for the failure of NBA, LBA, and CBA to alter PCa cellular functions could be that CBA, NBA, and LBA block TRPM4 conductivity insufficiently in androgen insensitive PCa cells [34].

Up to now, none of these TRPM4 inhibitors was studied in CRC cells. TRPM4 ion conductivity was recently shown to contribute to the regulation of the cell cycle and viability of CRC cell line, HCT116 [11]. Therefore, we hypothesized that the blockage of TRPM4 conductivity with CBA, LBA, and NBA would decrease viability and alter the cell cycle in HCT116 cells through the inhibition of TRPM4 current. In this study, we demonstrate for the first time that CBA, NBA, and LBA almost entirely block TRPM4 currents in CRC cells. Consequently, we can show that NBA, the most potent blocker, alters CRC cells proliferation and induces a shift in the cell cycle, specifically through TRPM4. This is the first pharmacological approach showing that small molecule inhibitor, through inhibition of TRPM4 conductivity, alters cancer hallmark functions.

## 2. Materials and Methods

### 2.1. Cell Culture

Human colorectal carcinoma cell line HCT116 was a gift from Karen Rother [35]. HCT116-TRPM4-knock-out clones (T4KO 1 and T4KO 2) were generated as described previously [11].

HCT116 and T4KO cells were cultivated in McCoy’s 5A (Modified) Medium with GlutaMAX (Gibco, Waltham, MA, USA) supplemented with 10% FBS (Gibco). Dukes’ type B colorectal adenocarcinoma cell line LS180 was purchased from ATCC and cultivated in MEM (Gibco) supplemented with 2 mM L-glutamine (Gibco), 1% NEAA (Gibco), and 10% FBS (Gibco). Dukes’ type D colorectal adenocarcinoma (derived from metastatic site) cell line Colo205 and Dukes’ type C colorectal adenocarcinoma cell line HCT15 were purchased from ATCC and cultivated in RPMI (Gibco) supplemented with 10% FBS (Gibco).

### 2.2. Electrophysiology

Whole-cell patch clamp experiments were performed in HCT116 cells at 22–25 °C. Every 2 s, voltage ramps of 50 ms, spanning from −100 to +100 mV from a holding potential of 0 mV, were delivered using the HEKA EPC-10 amplifier. Currents were acquired, digitized, recorded, and analyzed with a HEKA EPC-10 amplifier, HEKA Patchmaster v2 × 53, and Igor Pro 6.37 software (WaveMetrics, Portland, OR, USA). The liquid junction potential of 10 mV was determined using patch master’s power tools ([36]) and corrected. Currents at −80 mV and +80 mV were normalized to cell capacitance and plotted versus time. The bath solution contained 140 mM NaCl, 0.5 mM CaCl_2_, 3 mM MgCl_2_, and 10 mM HEPES. Osmolarity of the bath solution was adjusted to 300 mOsm with glucose, and pH was adjusted to 7.2 with NaOH. The internal pipette solution contained 140 mM Cs-glutamate, 10 mM EDTA, 10 mM HEPES, and 8 mM NaCl. WEBMAXC STANDARD software (version 7/3/2009, UC Davis, Davis, CA, USA) [37] was used to calculate the free Mg^2+^ (3 mM) and Ca^2+^ (10 µM) in the internal pipette solution. Only patch pipettes with a resistance of 2–3 MΩ were used for the measurements.

### 2.3. Drug Treatment

Three TRPM4 blockers were evaluated with patch-clamp technique and cellular assays: 4-chloro-2-(2-chlorophenoxy) acetamido benzoic acid (CBA), 4-chloro-2-(2-(4-chloro-2-methylphenoxy) propanamido) benzoic acid (LBA), and 4-chloro-2-(1-naphthyloxyacetamido) benzoic acid (NBA). The inhibitors were dissolved in dimeythylsulfoxide (DMSO). Chemical structures for CBA, LBA, and NBA are given in Figure 1A–C. For viability assay, cell cycle evaluation, and proliferation assay, DMSO at a concentration of 0.05% was used as a control. The DMSO in each sample was adjusted to 0.05%.

### 2.4. Viability Assay

Cells were seeded in triplicates on 96-well plates (HCT116 5 × 10^3^ cells per well, Colo205, HCT15, LS180 2.5 × 10^3^ cells per well) in a standard growth media. Cells were seeded 24 h before the start of the measurement to allow them to adhere to the bottom of the plate. Viability assay was performed with the use of RealTime-Glo MT Cell Viability Assay kit (Promega, Madison, WI, USA) according to the manufacturer’s instructions. Using a Tecan Spark™ (Tecan, Männedorf, Switzerland) 10 M multimode microplate reader, luminescence intensity was monitored every hour over a 48 h period. Data for each measurement were normalized to the measurement at the first hour. Statistical significance was analyzed using a one-way ANOVA with GraphPad Prism (GraphPad 9.1.1 Software, GraphPad Software, San Diego, CA, USA) software.

### 2.5. Proliferation Assay

Cell proliferation was evaluated with xCELLigence^®^ E-Plates on the xCELLigence^®^ RTCA DP system (ACEA Biosciences, San Diego, CA, USA). In this label-free assay, changes in impedance are recorded as the cells adhere to the plate surface. The plates were set up following the standard protocol of the manufacturer. Then, 3.5 h after the seeding of the cells, 100 µL of the media was replaced with 100 µL of CBA, LBA, or NBA at different concentrations, or DMSO as a control and measurement was continued. The proliferation of the cells was recorded over 70 h. The data were normalized to the measurement before the addition of the inhibitors or DMSO. Statistical significance was analyzed using a one-way ANOVA with GraphPad Prism (GraphPad 9.1.1 Software) software.

### 2.6. Cell Cycle Analysis

Propidium iodide (PI) staining was performed to determine cell cycle distribution. Cells were pre-seeded in 6-well plates and cultivated in a standard growth media. After 24 h, cells were treated with NBA or DMSO control for another 24 h. The cells were then fixed in 66% ethanol and incubated for at least 4 h at 4 °C. After incubation, cells were washed with 1× PBS, resuspended in 200 μL 1× PI + RNase Staining Solution (Abcam, ab139418, Abcam, Cambridge, United Kingdom) and incubated at 37 °C in the dark for 30 min. Following incubation, cell cycle was detected with LSR II BD flow cytometer and FACSDiva software (BD FACSDiva v9.0, BD Biosciences, Franklin Lakes, NJ, USA). Cell cycle data analysis and statistics were performed with FlowJo10 (FlowJo, LLC., BD Biosciences, Franklin Lakes, NJ, USA) and GraphPad Prism (GraphPad 9.1.1 Software) software. Statistical significance was analyzed with one-way ANOVA.

## 3. Results

### 3.1. Novel TRPM4 Inhibitors Block Endogenous TRPM4 Currents in CRC Cells

Recently, three novel TRPM4 inhibitors CBA, LBA, and NBA (Figure 1A–C) have been described and tested for their ability to block endogenous TRPM4 currents in PCa cells [34]. In the present study, we evaluated these three inhibitors in the CRC cell line, HCT116, and assessed their IC_50_ values in this cancer model (Figure 1D–I). TRPM4-specific currents were evaluated with the whole-cell patch clamp method upon activation of cells with 10 µM Ca^2+^ in the patch pipette (Figure 1D–F). After the current development (280 s), 10 µM of either CBA, NBA, or LBA was applied for 120 s. Afterwards, a standard bath solution was applied to wash off the inhibitors. The current-voltage (IV) relationship curve presents currents before (t = 276 s) and during (t =396 s) the application of the inhibitor (Figure 1D–F). All three inhibitors were able to almost completely (91.8–98.5% of the current) block the TRPM4-characteristic current (Figure 1D–F). This is interesting to note as, in PCa cells, neither of these inhibitors was able to completely block the current [34]. Additionally, the current was not restored after the wash-out of inhibitors, demonstrating that in CRC cells, the current inhibition is non-reversible. This is also in contrast to the findings in prostate cancer cells [34]. The IC_50_ values were calculated to be 1.18 µM for CBA, 0.12 µM for NBA, and 1.84 µM for LBA (Figure 1G–I). This is in good agreement with previously recorded currents in HEK293 TRPM4 overexpressing cells and PCa cells [19,34].

### 3.2. Novel TRPM4 Inhibitors Show No Effect on HCT116 Viability

**Figure 2:**
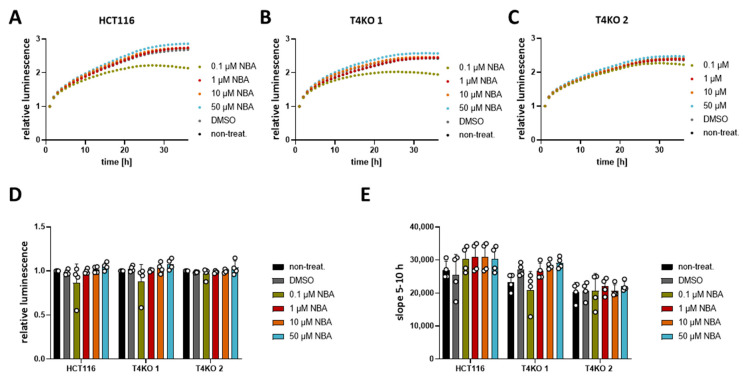
Viability of HCT116 and T4KO cell lines after treatment with NBA. Cell viability in HCT116, T4KO 1, and T4KO 2 cells was evaluated using a RealTime-Glo MT assay. Cells were treated with 0.1 µM, 1 µM, 10 µM, 50 µM NBA, or DMSO control. Four independent experiments were performed. (**A**) Mean of relative luminescence was plotted versus time for HCT116 cells. (**B**) Same for T4KO 1 cells. (**C**) Same for T4KO 2 cells. (**D**) Scatter plot and bar diagram of data (mean + SD) at 24 h from four independent experiments in (**A**–**C**). (**E**) Scatter plot and bar diagram of slope steepness between 5 and 10 h (mean + SD) from data in (**A**–**C**). One-way ANOVA was used to determine statistical significance in (**D**,**E**).

As previously shown, TRPM4 conductivity regulates the viability of CRC cells [11]. Upon TRPM4 knock-out, cell viability was decreased. Therefore, here, we tested different concentrations of CBA, LBA, and NBA on the viability of HCT116 cells and the two TRPM4-knock-out cell lines. First, analysis of the viability curves of HCT116 and T4KO without any treatment confirmed the previous findings with decreased viability of the TRPM4 knock-out cell lines, T4KO 1 and T4KO 2, compared to HCT116, although with a less pronounced decrease than the one observed in the original study (Figure S1A,B). However, this is to be expected since we started the original measurements directly after cells were seeded and the curves were normalized to the first hour. Here, cells were pre-seeded 24 h before starting the measurement; hence, the curves had to be normalized to a later point. Next, we analyzed the slope of the viability curve between the 5th and 10th hour, which indicates how fast cells are dividing/growing (Figure S1C). Slope analysis is independent of the normalization point, and it shows clearly that T4KO 1 and T4KO 2 cell lines displayed lower viability. However, treatment of HCT116 or T4KO 1 and T4KO 2 with the TRPM4 inhibitors showed no effect on the viability (Figure 2 for NBA and Figures S2 and S3 for CBA and LBA, respectively), except form 0.1 µM NBA. However, this effect was unspecific to TRPM4, as the viability was decreased not only in HCT116 but also in T4KO 1 cells. Moreover, this effect was observed only in one measurement. To summarize, despite the ability of NBA, CBA, and LBA to block TRPM4 currents, no effect on viability was detected in HCT116 cells.

### 3.3. TRPM4 Inhibitors Decrease the Proliferation of HCT116 Cells

**Figure 3:**
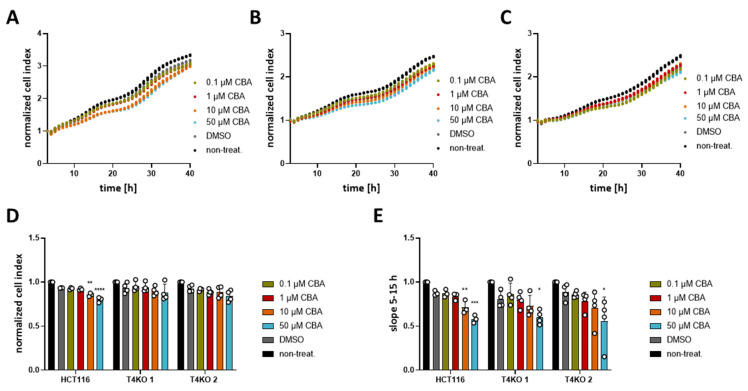
Effect of CBA on HCT116 cells’ proliferation. Cell proliferation was determined with an xCELLigence^®^ system. Cells were treated with 0.1 µM, 1 µM, 10 µM, 50 µM CBA, or DMSO control. A total of 3–4 independent experiments were performed. (**A**) Mean of cell index was plotted versus time for HCT116 cells. (**B**) Same as (**A**) for T4KO 1 cells. (**C**) Same as (**A**) for T4KO 2 cells. (**D**) Scatter plot and bar diagram of data (mean + SD) at 24 h from the experiment in (**A**–**C**). (**E**) Scatter plot and bar diagram of slope steepness between 5 and 15 h (mean + SD) from data in (**A**–**C**). One-way ANOVA was used to determine statistical significance (* *p* < 0.05, ** *p* < 0.005, *** *p* < 0.0005, **** *p <* 0.0001) in (**D**,**E**).

**Figure 4:**
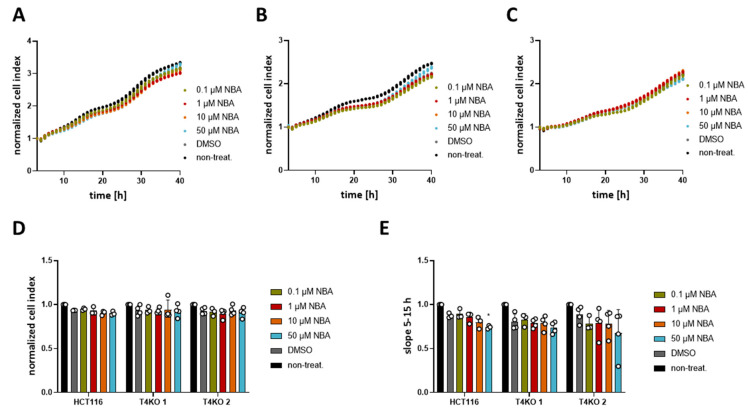
Effect of NBA on HCT116 cells’ proliferation. Cell proliferation was determined with an xCELLigence^®^ system. Cells were treated with 0.1 µM, 1 µM, 10 µM, 50 µM NBA, or DMSO control. A total of 3–4 independent experiments were performed. (**A**) Mean of cell index was plotted versus time for HCT116 cells. (**B**) Same as (**A**) for T4KO 1 cells. (**C**) Same as (**A**) for T4KO 2 cells. (**D**) Scatter plot and bar diagram of data (mean + SD) at 24 h from the experiment in (**A**–**C**). (**E**) Scatter plot and bar diagram of slope steepness between 5 and 15 h (mean + SD) from data in (**A**–**C**). One-way ANOVA was used to determine statistical significance (* *p* < 0.05) in (**D**,**E**).

Even though the TRPM4 inhibitors failed to alter the viability, we decided to test CBA, NBA, and LBA for their ability to alter proliferation in an impedance-based assay. In contrast to the viability assay, in which the metabolic activity of cells is measured, here we measure changes in impedance as the cells adhere to the surface of the plate. Increased cell index reflects the number of cells; therefore, higher numbers suggest that cells proliferate more. In addition, we also analyzed the growth curve between the 5th and the 15h hour reflecting cell proliferation and between the 1st and the 3rd hour reflecting cell adhesion according to [38]. First, we analyzed the differences in proliferation between HCT116 and T4KO cells (Figure S4). Both normalized cell index and the slope between the 5th and 15th hour show that T4KO cells proliferate slower than HCT116. Compared to HCT116 parental cells, we find a slightly reduced cell adhesion in one of the KO clones that was not significant (Figure S4D). Next, we tested the effect of CBA, LBA, and NBA on proliferation (cell index and slope between the 5th and the 15h hour). Our results show that HCT116 cells treated with either 10 µM or 50 µM CBA displayed lower cell index after 24 h and decreased slope in comparison to the DMSO treated control (Figure 3). This effect appears to be dose dependent. However, we also observed a slight effect of CBA, especially at the concentration of 50 µM, on the slope in T4KO cells, although not as prominent as in the parental cells. This suggests that CBA might also target some other protein aside from TRPM4. NBA treatment of HCT116 cells did not affect the cell index at 24 h; however, it decreased the slope in a dose-dependent manner, and this decrease was statistically significant for cells treated with 50 µM NBA (Figure 4). No statistically significant difference was observed for the T4KO cells, suggesting that the effect of NBA on proliferation is specific to TRPM4. LBA at the concentration of 50 µM also decreased the growth speed of HCT116 cells; however, we also observed an effect of 50 µM LBA on the slope of T4KO cells (Figure S5).

In conclusion, the tested TRPM4 inhibitors affect HCT116 cell proliferation; however, the effect is not entirely specific to TRPM4, especially in the case of CBA and LBA. Given that the slope was decreased in HCT116 cells treated with 50 µM NBA but not in T4KO cells, we conclude that NBA decreases the proliferation of HCT116 cells specifically through TRPM4.

### 3.4. NBA Affects Cell Cycle in HCT116 Cells

**Figure 5:**
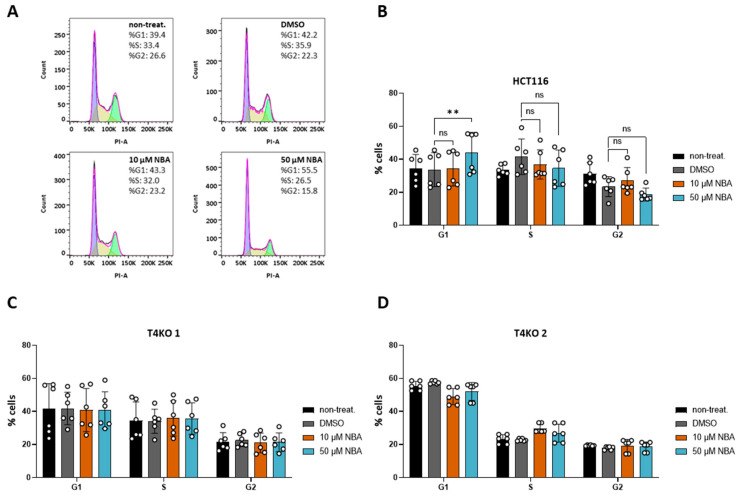
Effect of NBA on cell cycle in HCT116 and T4KO cells. FACS-based cell cycle analysis. The experiment was repeated four times with from one to two replicates in each experiment. (**A**) Representative histograms of PI staining in HCT116 without inhibitor treatment, treated with DMSO and treated with 10 µM and 50 µM NBA, with cell distribution in % for one specific measurement. (**B**) Scatter plot and bar diagram (mean + SD) for cell cycle distribution of HCT116 without inhibitor treatment, treated with DMSO and treated with 10 µM and 50 µM NBA. (**C**) Scatter plot and bar diagram (mean + SD) for cell cycle distribution of T4KO 1 without inhibitor treatment, treated with DMSO and treated with 10 µM and 50 µM NBA. (**D**) Scatter plot and bar diagram (mean + SD) for cell cycle distribution of T4KO 2 without inhibitor treatment, treated with DMSO and treated with 10 µM and 50 µM NBA. One-way ANOVA was used to determine statistical significance (** *p* < 0.005, ns—non significant) in (**B**–**D**).

Aside from the role of TRPM4 conductivity in HCT116 viability, we previously showed that the lack of TRPM4 affects cell cycle distribution in HCT116 [11]. Here, we decided to test NBA for its effect on cell cycle distribution due to its low IC_50_ value in the patch clamp measurements and specificity during the proliferation assay. We were able to reproduce findings from our previous study [11], as T4KO cell lines exhibited a significant shift in the cell cycle (Figure S6). Next, our results of the inhibitor treatment showed no effect of 10 µM NBA, while treatment with 50 µM NBA resulted in an increased number of cells G1 phase (Figure 5A,B). This is in line with the TRPM4-knock-out data. NBA had no effect on the T4KO cell lines, indicating that the effect of 50 µM NBA on HCT116 cells was specific to (the inhibition of) TRPM4 (Figure 5C,D). Taken together, this suggests that the TRPM4 regulation of the cell cycle is mediated by TRPM4 conductivity.

### 3.5. NBA Reduces the Viability of Colo205 Cells

**Figure 6:**
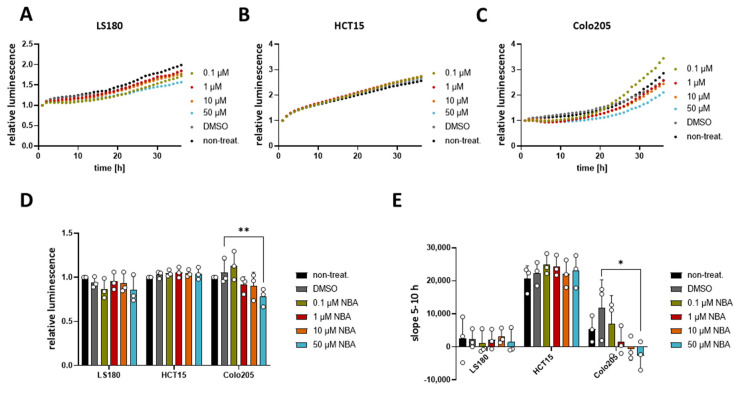
Effect of NBA on CRC cells’ viability. Cell viability in LS180, HCT15, and Colo205 cells was evaluated using a RealTime-Glo MT assay. Cells were treated with 0.1 µM, 1 µM, 10 µM, 50 µM NBA, or DMSO control. Three independent experiments were performed. (**A**) Mean of relative luminescence was plotted versus time for LS180 cells. (**B**) Same for HCT15 cells. (**C**) Same for Colo205 cells. (**D**) Scatter plot and bar diagram of data (mean + SD) at 24 h from three independent experiments in (**A**–**C**). (**E**) Scatter plot and bar diagram of slope steepness between 5 and 10 h (mean + SD) from data in (**A**–**C**). One-way ANOVA was used to determine statistical significance (* *p* < 0.05, ** *p* < 0.005) in (**D**,**E**).

Since NBA showed an effect on the cell cycle in HCT116, we decided to evaluate if this compound could affect the cell viability of other CRC cell lines. For this purpose, we used three different CRC cell lines LS180, HCT15, and Colo205, which all express TRPM4, although at different levels [11]. Our result showed that NBA had no effect on the viability of LS180 and HCT15 cell lines (Figure 6A–C), while it decreased the viability of Colo205 cells at a concentration of 50 µM (Figure 6C,D). Additionally, the analysis of the slope of the Colo205 viability curve revealed a dose-dependent response to the treatment (Figure 6E). Concentrations of 1 µM, 10 µM, and 50 µM NBA reduced the growth of Colo205 cells. The inhibition (23%) was statistically significant for 50 µM NBA. Notably, NBA inhibits the growth of Colo205, which is the CRC cell line with the highest TRPM4 expression and the cell line representing the most malignant Dukes’ D stage.

## 4. Discussion

The ion channel TRPM4 is expressed in many different types of tissues, but its role in physiology and pathophysiology is still poorly understood. TRPM4 has been associated with cardiovascular and immune disorders, as well as cancer [4,6,7,8,9,10,11,12,13,14,15]. One important limitation for the further understanding of the physiological and pathophysiological role of TRPM4 is the lack of potent and selective inhibitors [13,31,32]. Similar to other TRP channels, TRPM4 is mostly expressed on the cell surface. This fact makes TRPM4 an attractive drug target candidate, as drugs targeting TRPM4 would not need to enter the cell. Additionally, there is a need for specific TRPM4 inhibitors, which could be used on primary cell lines and animal models.

We have previously shown that TRPM4 is a novel potential biomarker candidate for colorectal cancer (CRC) [11]. Elevated TRPM4 protein expression levels are present in CRC tissues, characterized by high numbers of tumor buds and an increased percentage of infiltrative tumor border configuration. These characteristics are correlated with an increased frequency of vessel invasion and lymph node metastasis [11,27,28]. Importantly, on a cellular level, TRPM4 conducts large Na^+^ currents in CRC cells, and these currents play a role in the regulation of CRC cell viability and cell cycle [11]. HCT116 cells with TRPM4 knock-out show reduced proliferation and viability compared to parental cells; however, we cannot exclude that they underwent a genetic shift, adjusting them to the lack of TRPM4. Nevertheless, the fact that HCT116 T4KO cells show reduced proliferation and viability is important for the validation of novel TRPM4 inhibitors, as the inhibition of TRPM4 currents could potentially alter cellular functions and cancer hallmark functions in CRC cells. Here, we hypothesized that novel TRPM4 inhibitors would alter viability and cell cycle in HCT116 cells. These inhibitors, namely CBA, LBA, and NBA, were first tested in a patch clamp experiment for their potency to block endogenous TRPM4 currents in HCT116. Our results showed that CBA, LBA, and NBA block TRPM4 currents with IC_50_ values in a low micromolar range. Interestingly, these inhibitors act differently than in PCa cells. In contrast to current inhibition in PCa cells, in HCT116 cells, the TRPM4 current inhibition is non-reversible. Furthermore, in PCa, the inhibition of TRPM4 current was not complete [34], while in HCT116, we could achieve inhibition close to 100% (range of 91.8–98.5%). These characteristics can have an impact on the potential of these inhibitors to alter cellular functions via inhibition of the TRPM4 current. Our results show no difference in the viability of HCT116 cells upon application of neither of the inhibitors. In our previous work, we showed that TRPM4 conductivity clearly contributed to the regulation of HCT116 cells’ viability [11]. Therefore, it is unexpected that we see no change in HCT116 cells’ viability upon the treatment with TRPM4 inhibitors. Despite the lack of inhibitory effect on HCT116 cells’ viability, we decided to further test the blockers for their potency to alter HCT116 cells’ proliferation in an impedance-based assay. Our results show that all three inhibitors slowed the proliferation of HCT116 at the concentration of 50 µM. We additionally observed an effect of 10 µM CBA. However, 50 µM CBA and LBA also decreased the proliferation of T4KO cells, although less than in parental cells, which indicates that CBA and LBA potentially interact not only with TRPM4. NBA, on the other hand, decreased the proliferation (slope) only of the parental cells and not the T4KO cells indicating that the inhibition is TRPM4 specific. Additionally, we show that NBA affects cell cycle distribution in these cells. Upon treatment with 50 µM NBA, we observed that more cells were in the G1 phase of the cell cycle. This resembled the effect of TRPM4 knock-out, as the cell cycle distribution pattern upon treatment with 50 µM NBA was similar to the T4KO clones. NBA treatment of the two T4KO cell lines did not change the cell cycle distribution, suggesting that the observed effect of the inhibitor on the cell cycle is specific to TRPM4. In addition, we tested NBA in other CRC cell lines. Our results show that while NBA had no effect on the viability of HCT15 and LS180 cell lines, it inhibited the viability of Colo205 cells. Notably, NBA showed an effect in these cells, as Colo205 is the cell line that expresses TRPM4 on the highest level [11]. Therefore, it is possible that when TRPM4 is highly expressed, NBA inhibits viability via modulation of TRPM4 function. Given the fact that TRPM4 is upregulated in tumor buds of CRC, markers of disease progression, this can become interesting for the development of new therapies. Potentially, this provides an opportunity to target only cells in which TRPM4 expression is upregulated.

NBA is more specific to TRPM4 than other available TRPM4 inhibitors [19], such as 9-phenantrol, which interacts with the TMEM16A channel [39], and transient outward, rapid rectifier K^+^ channels [40], or glibenclamide, which also inhibits K_ATP_ channels [33]. Recently, a new monoclonal antibody, M4P, against TRPM4 was developed and was shown to specifically inhibit TRPM4 currents. M4P could contribute to research on TRPM4 in disease models [41]. However, small TRPM4 inhibitors will possibly have an advantage because of their lower price and smaller size resulting in the ability to diffuse through the membrane. So far, the binding sites to TRPM4 protein of CBA, LBA, and NBA are speculative. Currently, research is conducted in order to describe NBA, CBA, and LBA in more detail [42].

The discovery of new, potent, and selective TRPM4 inhibitors will allow us to better understand the role of TRPM4 in the pathophysiology of CRC by enabling us to study TRPM4 in animal models of CRC. Ultimately, this could lead to the development of drugs targeting TRPM4 in CRC therapy. In the nervous system, TRPM4 contributes to the progression of multiple sclerosis, stroke, spinal cord injury, and head injury [20,43,44]. Moreover, mutations in TRPM4 have been found in cardiovascular diseases, including bundle-branch block and Brugada syndrome [45]. In addition, aside from CRC, aberrant expression of TRPM4 has been linked to cancers arising from prostate, liver, bladder, breast, and large B cells [6,11,12,14,24,25,46,47,48]. Therefore, TRPM4 inhibitors such as NBA could potentially become useful tools for the study of TRPM4 in various diseases.

## 5. Conclusions

In conclusion, we here show that the new compound NBA inhibits TRPM4 currents with a low IC_50_ value, decreases proliferation, and induces a shift in the cell cycle. Furthermore, NBA decreases the viability of Colo205 cells, which express high levels of TRPM4. This makes NBA a promising candidate for further evaluation as a specific TRPM4 inhibitor in other cellular systems and disease models.

## Data Availability

Data depository will be made available at Zenodo.

## Supplementary Materials

The following are available online at https://www.mdpi.com/article/10.3390/cancers13215400/s1, Figure S1: Viability of T4KO cell lines is decreased in comparison to the parental cell line, HCT116, Figure S2: Viability of HCT116 and T4KO cell lines after treatment with CBA, Figure S3: Viability of HCT116 and T4KO cell lines after treatment with LBA, Figure S4: Proliferation of HCT116 and T4KO cell lines, Figure S5: Effect of LBA on HCT116 cells’ proliferation, Figure S6: Cell cycle distribution in HCT116 and T4KO cell lines.
