# Supplementary material for: Investigation of Novel Small Molecular TRPM4 Inhibitors in Colorectal Cancer Cells
Source: Cancers (Basel). 2021 Oct 28;13(21):5400. doi: 10.3390/cancers13215400 (PMC8582472; doi:10.3390/cancers13215400)
Supplement: Supplementary file 1 [file cancers-13-05400-s001.zip › cancers-1374645-supplementary.pdf]

cancers

MDPI

Supplementary Materials: Investigation of Novel Small Molecular TRPM4 Inhibitors in Colorectal Cancer Cells

Paulina Stokłosa, Anna Borgström, Barbara Hauert, Roland Baur and Christine Peinelt

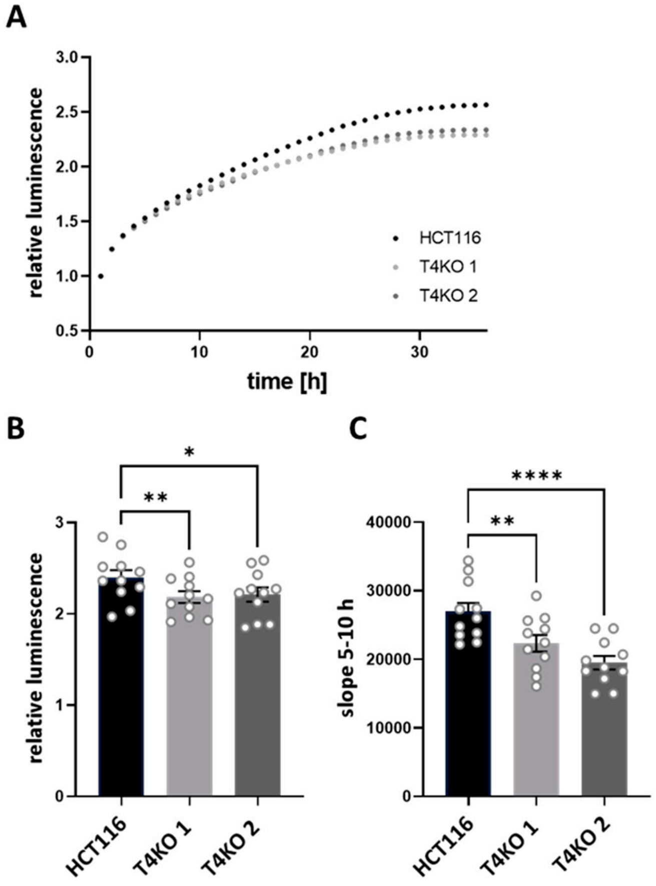

Figure S1. Viability of T4KO cell lines is decreased in comparison to the parental cell line, HCT116. Analysis of cell viability in HCT116, T4KO 1 and T4KO 2 cells. Data were pooled from viability experiments (non- treat. control) in Figure 1 A–C (n = 4), Figure S2 A-C (n = 4) and Figure S3 A–C (n = 3) for HCT116, T4KO 1 and T4KO 2. The overall sample size is 11. Cell viability was evaluated using RealTime Glo MT assay. (**A**) Mean of relative luminescence was plotted versus time for HCT116, T4KO 1 and T4KO 2 cells. (**B**) Scatter plot and bar diagram of data (mean + SD) at 24 h (**C**) Scatter plot and bar diagram of slope steepness between 5–10 h (mean + SD) from data in (**A**), (**B**), (**C**). One-way ANOVA was used to determine statistical significance in (* $p < 0.05$, ** $p < 0.005$, *** $p < 0.0005$, **** $p < 0.0001$) in B and C.

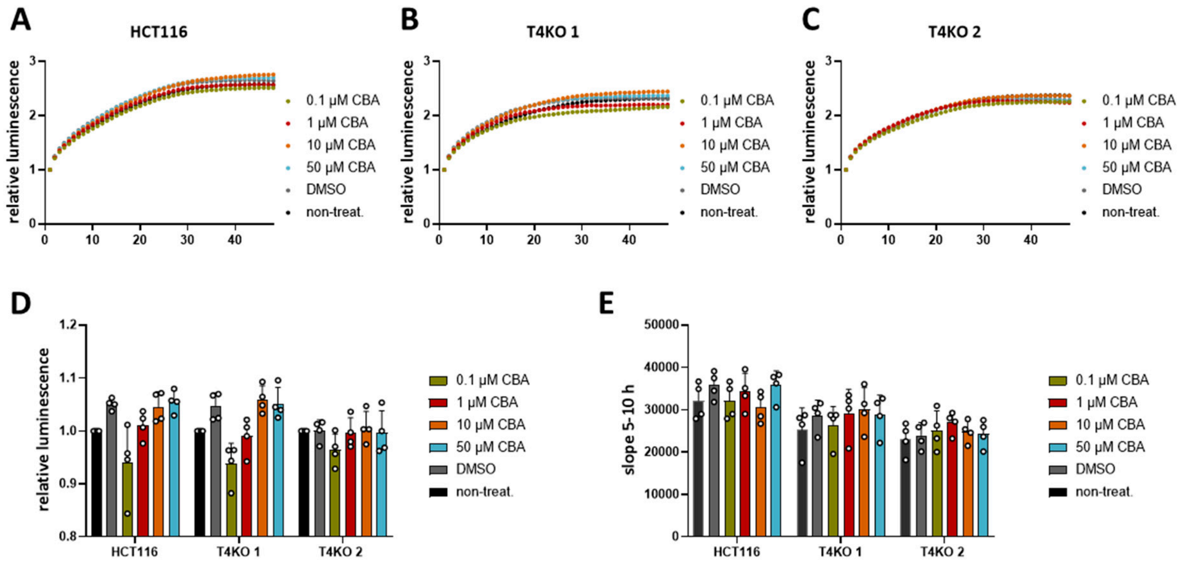

Figure S2. Viability of HCT116 and T4KO cell lines after treatment with CBA. (**A**) Mean of relative luminescence was plotted versus time for HCT116 cells. (**B**) Same for T4KO 1 cells. (**C**) Same for T4KO 2 cells. (**D**) Scatter plot and bar diagram of data (mean + SD) at 24 h from four independent experiments in (**A**), (**B**), (**C**). (**E**) Scatter plot and bar diagram of slope steepness between 5–10 h (mean + SD) from data in (**A**), (**B**), (**C**).

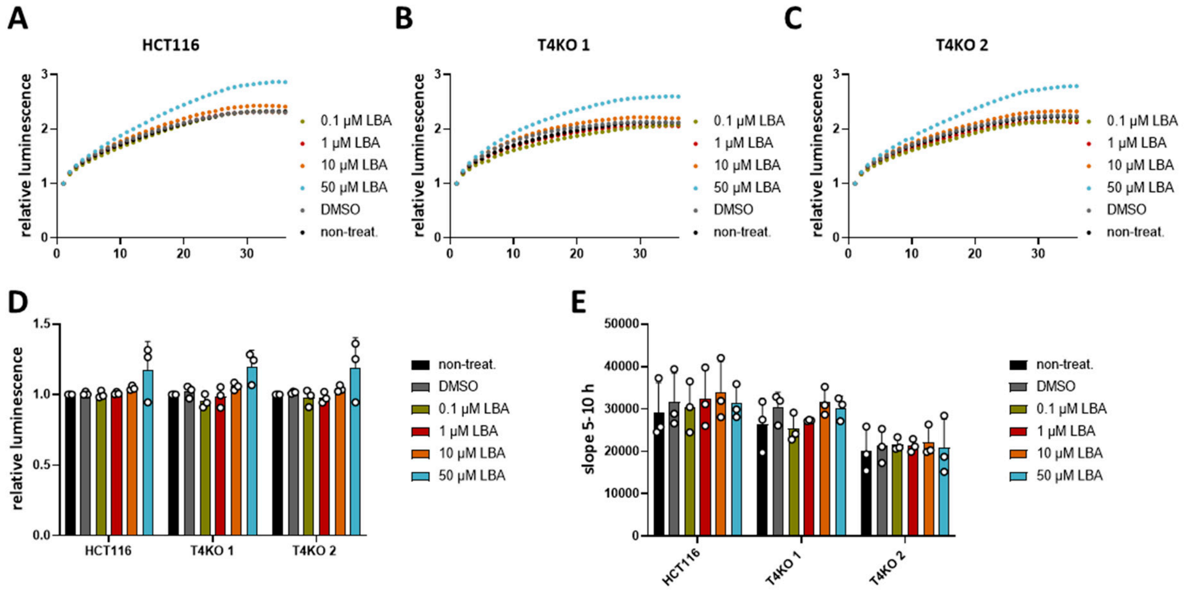

Figure S3. Viability of HCT116 and T4KO cell lines after treatment with LBA. Cell viability in HCT116, T4KO 1 and T4KO 2 was evaluated using a RealTime-Glo MT assay. Cells were treated with 0.1 µM, 1 µM, 10 µM, 50 µM LBA or DMSO control. Three independent experiments were performed. (**A**) Mean of relative luminescence was plotted versus time for HCT116 cells. (**B**) Same for T4KO 1 cells. (**C**) Same for T4KO 2 cells. (**D**) Scatter plot and bar diagram of data (mean + SD) at 24 h from three independent experiments in (**A**), (**B**), (**C**). (**E**) Scatter plot and bar diagram of slope steepness between 5–10 h (mean + SD) from data in (**A**), (**B**), (**C**).

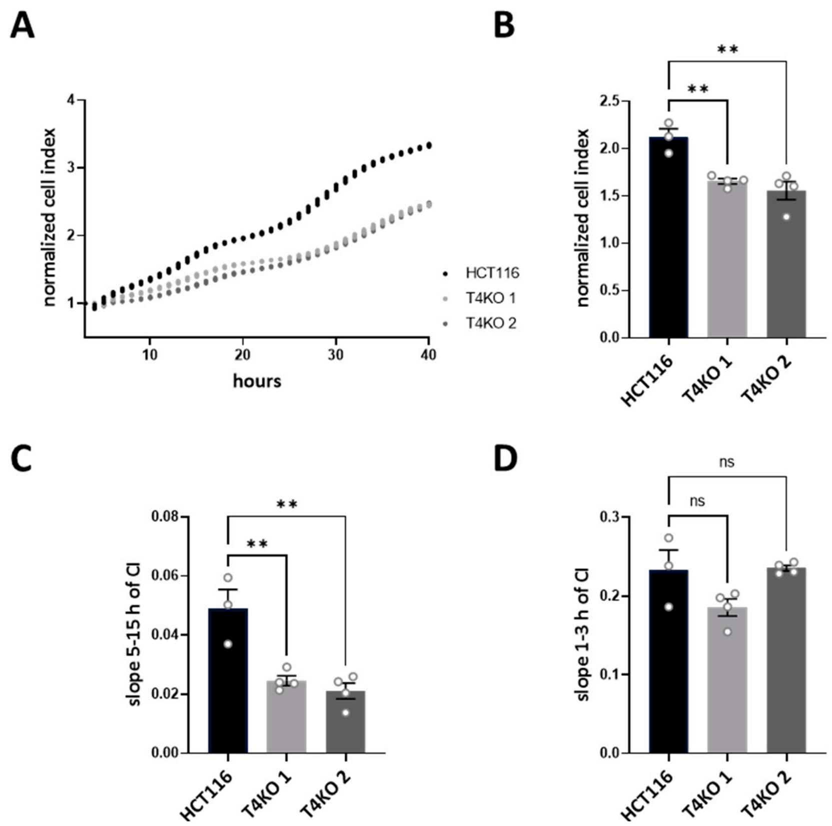

Figure S4. Proliferation of HCT116 and T4KO cell lines. Cell proliferation was determined with an xCELLigence® system. Three to four independent experiments were performed. (**A**) Mean of normalized cell index was plotted versus time for HCT116, T4KO 1 and T4KO 2. (**B**) Scatter plot and bar diagram of data (mean + SD) at 24 h from the experiment in (**A**). (**C**) Scatter plot and bar diagram of slope steepness (mean + SD) between 5–15 h from data in (**A**). (**D**) Scatter plot and bar diagram of slope steepness (mean + SD) 1–3 h from data in (**A**). One-way ANOVA was used to determine statistical significance (*$p < 0.05$, **$p < 0.005$) in D and E.

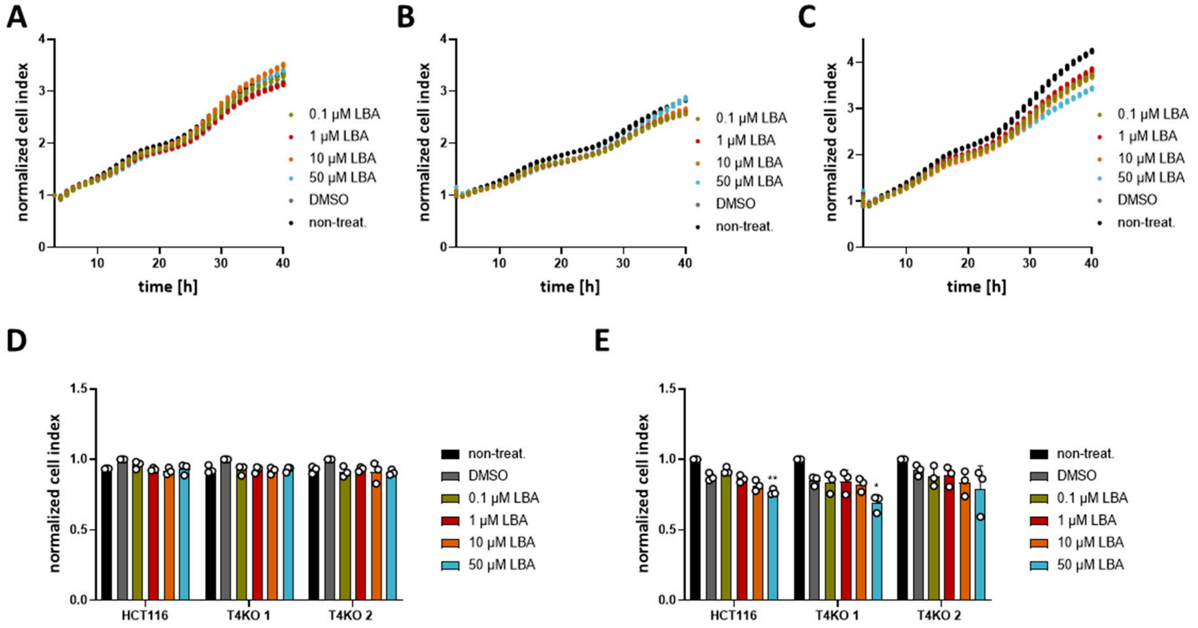

Figure S5. Effect of LBA on HCT116 cells' proliferation. Cell proliferation was determined with an xCELLigence® system. Cells were treated with 0.1 µM, 1 µM, 10 µM, 50 µM LBA or DMSO control. Three independent experiments were performed. (**A**) Mean of cell index was plotted versus time for HCT116 cells. (**B**) Same as (**A**) for T4KO 1 cells. (**C**) Same as

(A) for T4KO 2 cells. (**D**) Scatter plot and bar diagram of data (mean + SD) at 24 h from the experiment in (**A**), (**B**), (**C**). (**E**) Scatter plot and bar diagram of slope steepness between 5–15 h (mean + SD) from data in (**A**), (**B**), (**C**).

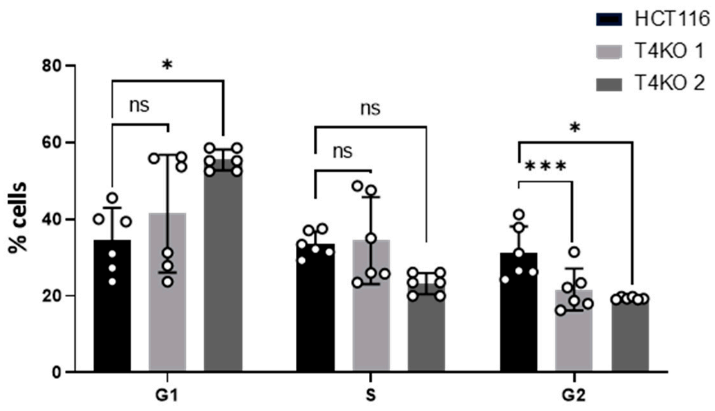

Figure S6. Cell cycle distribution in HCT116 and T4KO cell lines. FACS-based cell cycle analysis. Experiment was repeated three times with two replicates in each experiment. Scatter plot and bar diagram (mean + SD) for cell cycle distribution of HCT116, T4KO 1 and T4KO 2 cells (* $p < 0.05$, *** $p < 0.0005$, ns—non significant).
